# Applications of fractional lower order S transform time frequency filtering algorithm to machine fault diagnosis

**DOI:** 10.1371/journal.pone.0175202

**Published:** 2017-04-13

**Authors:** Junbo Long, Haibin Wang, Daifeng Zha, Peng Li, Huicheng Xie, Lili Mao

**Affiliations:** 1 College of Electronic and Engineering Jiujiang University, Jiujiang, China; 2 College of Information Science and Engineering Technology Jiujiang University, Jiujiang, China; 3 College of Science Jiujiang University, Jiujiang, China; Chongqing University, CHINA

## Abstract

Stockwell transform(ST) time-frequency representation(ST-TFR) is a time frequency analysis method which combines short time Fourier transform with wavelet transform, and ST time frequency filtering(ST-TFF) method which takes advantage of time-frequency localized spectra can separate the signals from Gaussian noise. The ST-TFR and ST-TFF methods are used to analyze the fault signals, which is reasonable and effective in general Gaussian noise cases. However, it is proved that the mechanical bearing fault signal belongs to Alpha(*α*) stable distribution process(1 < *α* < 2) in this paper, even the noise also is *α* stable distribution in some special cases. The performance of ST-TFR method will degrade under *α* stable distribution noise environment, following the ST-TFF method fail. Hence, a new fractional lower order ST time frequency representation(FLOST-TFR) method employing fractional lower order moment and ST and inverse FLOST(IFLOST) are proposed in this paper. A new FLOST time frequency filtering(FLOST-TFF) algorithm based on FLOST-TFR method and IFLOST is also proposed, whose simplified method is presented in this paper. The discrete implementation of FLOST-TFF algorithm is deduced, and relevant steps are summarized. Simulation results demonstrate that FLOST-TFR algorithm is obviously better than the existing ST-TFR algorithm under *α* stable distribution noise, which can work better under Gaussian noise environment, and is robust. The FLOST-TFF method can effectively filter out *α* stable distribution noise, and restore the original signal. The performance of FLOST-TFF algorithm is better than the ST-TFF method, employing which mixed *MSEs* are smaller when *α* and generalized signal noise ratio(*GSNR*) change. Finally, the FLOST-TFR and FLOST-TFF methods are applied to analyze the outer race fault signal and extract their fault features under *α* stable distribution noise, where excellent performances can be shown.

## Introduction

The fault signal received by the sensors is non-stationary when the rotating machinery bearing break down, and time-frequency representation is a effective method to analyze the non-stationary signal[1–3]. Recently, several time-frequency methods have been applied to fault signal analysis[4–7]. The fault feature extraction algorithm based on short-time Fourier transform (STFT) time-frequency representation and non-negative matrix factorization method were proposed in [4]. Guoqi etc. proposed a joint time-frequency distribution method which combined Wigner-Ville time-frequency distribution with empirical mode decomposition[5], the method could effectively reduce the cross-term interference, and which was used in the rotating machinery fault signal analysis. Several time frequency methods were introduced in [6], and the application comparisions of the methods to fault signal analysis were summarized. Stockwell proposed S transform(ST) in 1996[7]. ST is a linear time-frequency distribution methods, and has good frequency resolution. Recently, ST time-frequency method has been widely applied to mechanical fault signal analysis[8–11]. Guo Yuanjin et al. applied ST method for feature extraction of the bearing fault signals, they verified the method could better extract impact characteristics, its performance advantage was reflected by comparing with STFT and wavelet transform[8]. Whereafter, http://www.youdao.com/w/whereafter/javascript:void(0);they put forward an improved S transform time-frequency method based on singular value decomposition in [9], which applied inverse S transform to extract impact feature of the fault signal, and better realized the fault diagnosis. A new detection method based on S transform and zero space was proposed in [10], and which was applied to bearing fault signal detection. A new method employing morphological wavelet and S-transform was presented in [11], which had less computational efforts, and could analyze fault signal online. Recently, A time frequency filtering method which take advantage of ST time-frequency localization and inverse ST were proposed for data-adaptive filter, and the methods were applied to analyze the earthquake data[12–13]. A time-frequency filtering method employing normalized window S transform and TT transform were proposed in [14], which were used to filter out high frequency noise and random noise in radar echo signal. An adaptive time-frequency filtering method based on generalized S-transform was proposed in [15], the method constructed a new adaptive time-frequency filtering factor, and was applied to filter out noises and retrieve LFM signals.

The mentioned methods in [7–15] are based on Gaussian hypothesis, and second order statistics is used in the methods. In most cases, Gaussian hypothesis is reasonable and effective, but in some special cases, probability density function of the mechanical bearing fault signal and the noise have an obvious trail. The signal and noise are non-stationary and non-Gaussian process, and belong to *α* stable distribution[16–19]. When *α* = 2, they belong to Gaussian distribution, and when 0 < *α* < 2, they are low-order *α* stable distribution. In *α* stable distribution environment, the performance of the mentioned methods in [7–15] degenerates when Gaussian model is employed to analyze the non-Gaussian signals. Hence, the theoretical model and method based on the fractional lower order can be used for the cases. Recently, *α* stable distribution was used to describe the machinery fault signal in [17]. A support vector machine algorithm based on *α* stable distribution was proposed in [18], which was applied to the mechanical fault analysis, and it was proved that the modified method could effectively improve learning and convergence speed of the samples. Gang Yu et al. further confirmed non-Gaussian characteristics of the machinery fault signal, and proposed a new signal detection method based on *α* stable distribution parameter and histogram for mechanical fault diagnosis, whose performance was better than the traditional method based on Gaussian model. In addition, L Zhang and the others proposed the fractional-order modeling and filtering techniques in [20–21], which have been applied in state-of-charge estimation and parameter identification of ultracapacitor.

In *α* stable distribution environment, the time frequency representation methods based on ST in literature [7–11] degrade, and even fail. Therefore, we combine the traditional S transform with fractional lower order moment, and propose a fractional lower order S transform(FLOST) algorithm and inverse fractional lower order S transform(IFLOST) algorithm. The corresponding fractional low order ST time frequency representation(FLOST-TFR) method can effectively demonstrate time frequency distribution of the signal under *α* stable distribution noise. Hence, the FLOST-TFR method provides an approach for the special time frequency analysis cases. IFLOST is inverse transform of FLOST, which provides a computational efficient way to restore the original signal from its time frequency distribution when the undesired parts are removed.

Similarly, the time frequency filtering methods in [7–11] degenerate, even fail. Therefore, a novel fractional lower order S transform time frequency filtering(FLOST-TFF) algorithm is proposed based on the proposed FLOST and IFLOST methods in this paper. Firstly, time-frequency distribution of the signal is obtained employing FLOST-TFR algorithm, and the effective signal which is clustered by energy is separated from *α* stable distribution noise. Finally, we apply IFLOST method to restore the original signal. Simulation results show that fractional lower order S transform time frequency algorithm can better work under Gaussian noise and *α* stable distribution noise environment, which is robust, and its performance is better than the existing S transform time frequency method. The proposed FLOST-TFF method can effectively separate out time frequency spectrum of the signal from *α* stable distribution noise, and restores the original signal. Mixed mean square error (MSE) of the FLOST-TFF method is significantly lower than that of the existing ST-TFF method under different characteristics index *α* and generalized signal noise ratio *GSNR*. Especially, the performance advantage of the FLOST-TFF method is more obvious when *GSNR* is low or *α* is small. Finally, we apply the proposed FLOST-TFR and FLOST-TFF algorithms to analyze the mechanical bearing outer race fault signals, the results show that the methods can better extract fault characteristics of the bearing fault signals, and restore the original signals under *α* stable noise environment.

In this paper, the improved S transform time-frequency representation and S transform time-frequency filtering methods based on fractional lower statistical moment are proposed for machine fault diagnosis. The paper is structured in the following manner. *α* stable distribution and the bearing fault signals are introduced in section 2. The modified fractional lower order S transform time-frequency representation and its inverse transform method are introduced in section 3, and the improved fractional lower order S transform time-frequency filtering method is demonstrated in section 4. Simulation comparisons with the conventional method based on ST are performed to demonstrate justifiability of the proposed methods based on FLOST, and the simulations of the outer race fault signals diagnosis are presented in section 5. Finally, the conclusions and future research are given in Section 6.

## *α* stable distribution and bearing fault signals

### *α* stable distribution

*α* stable distribution is a generalized Gaussian distribution, its characteristic function is defined as[22–24]

$$\varphi (t)=\exp\left\{j\mu t-\gamma {\left|t\right|}^{\alpha }[1+j\beta sign(t)\omega (\tau ,\alpha )]\right\}$$ (1)

**Fig 1:**
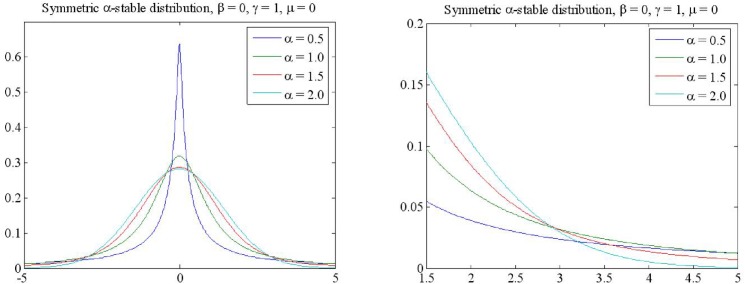
PDFs of *SαS* stable distribution in different *α*.

Where $\omega (\tau ,\alpha )=\{\begin{array}{cc} \tan(\alpha \pi /2) & if\;\alpha \neq 1\\ (2/\pi )\log\left|\tau \right| & \;if\;\alpha =1 \end{array}$, $sign(t)=\{\begin{array}{cc} 1 & t>0\\ 0 & t=0\\ -1 & \;t<0 \end{array}$, *α* is characteristic index, when 0 < *α* < 2, which is lower order *α* stable distribution, when *α* = 2, it belongs to Gaussian distribution, *β* is the symmetry coefficient, *γ* is the dispersion coefficient, *μ* is the location parameter, if *β* = 0, which is symmetric *α* stable(*SαS*) distribution. Fig 1 is probability density function(PDF) of *SαS* under *α* = 0.5, 1.0, 1.5 and 2.

### Bearing fault signals

The signal received by the vibration sensors is a non-stationary mixture when the rolling machinery bearing breaks down, which includes the fault signal, the other vibration signals, and the noises, etc. The experimental signals are selected from the case western reserve university data center in this paper[25], which are shown in S1, S2, S3 and S4 Mats. The fault points with 0.007 inches fault diameters are reinstalled into the test motor, the motor speed is 1797 RPM (revolutions per minute), and digital data is collected at 12,000 samples per second, the fault points are set up at inner race, outer race, and sphere race, respectively. Three acceleration sensors are placed to collect the signals in each fault point, which include base accelerometer (BA), drive end accelerometer(DE), and fan end accelerometer(FE), respectively.

**Fig 2:**
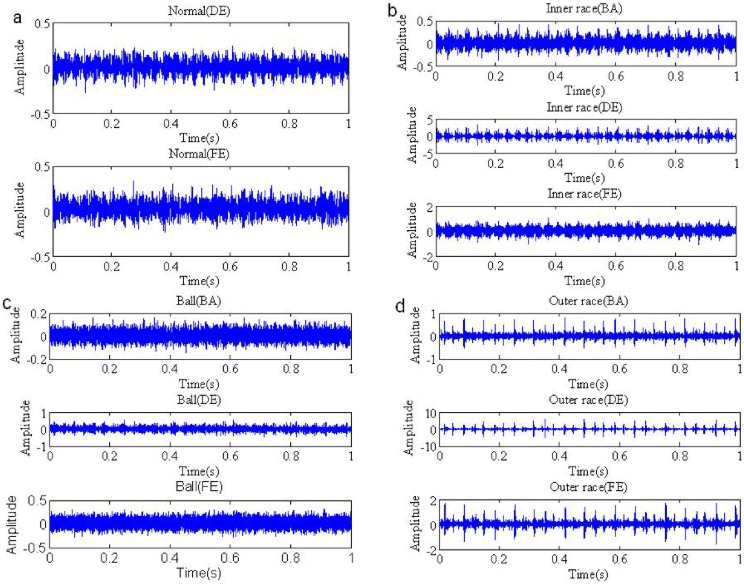
Waveforms of the bearing signals (a) Waveforms of normal signal in DE and FE (b) Waveforms of the inner race fault signals in BA, DE and FE (c) Waveforms of the ball fault signals in BA, DE and FE (d) Waveforms of the outer race fault signals in BA, DE and FE.

**Fig 3:**
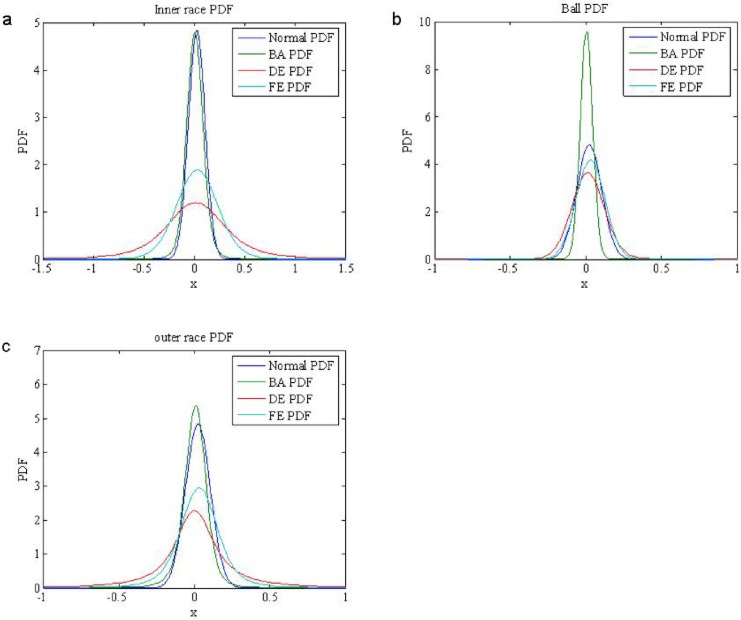
PDFs of the bearing fault signals (a) PDFs of inner race fault in DE, FE and BA (b) PDFs of ball fault in DE, FE and BA (c) PDFs of outer race fault in DE, FE and BA.

The time waveforms of the normal signal received by DE and FE accelerometers are shown in Fig 2(A). Fig 2B–2D are time waveforms of the inner race fault signals, the ball fault signals and the outer race fault signals, respectively. As can be seen from the figures, the waveforms of the normal signal in Fig 2(A) are similar with Gaussian process, but the waveforms of the fault signals in Fig 2B–2D have obvious pulsive character, which are a non-Gaussian process. To confirm that, We apply *α* stable distribution model to estimate the parameters of the normal signal and fault signals, the experimental results are shown in Table 1. The results demonstrate characteristic index of the normal signal *α* = 2, which belongs to Gaussian distribution, and the characteristic index of the fault signals as follows, inner race fault signals in BA, FE and DE are 1.0607, 1.1096 and 1.5435, respectively, the ball fault signals BA(*α* = 1.979), FE(*α* = 1.8697) and DE(*α* = 1.998), outer race fault signals BA(*α* = 1.6077), FE(*α* = 1.1096) and DE(*α* = 1.5435). Hence, it is proved that the bearing fault signals are non-Gaussian *α* stable distribution(1 < *α* < 2). Fig 3A–3C are PDFs of the normal signal and bearing fault signals, which show that PDFs of the bearing fault signals have a certain trailing.

**Table 1:** *α* stable distribution parameter estimations of the bearing fault signals.

parameters		*α*	*β*	*γ*	*μ*
**Normal**	**DE**	**2.000**	**-0.2863**	**0.0532**	**0.0121**
	**FE**	**2.000**	**1.000**	**0.0583**	**0.0236**
**Inner race**	**BA**	**1.7682**	**0.0872**	**0.0590**	**0.0062**
	**DE**	**1.4195**	**0.0155**	**0.2407**	**0.0175**
	**FE**	**1.8350**	**0.0322**	**0.1495**	**0.0291**
**Ball**	**BA**	**1.9790**	**0.0592**	**0.0293**	**0.0055**
	**DE**	**1.8697**	**0.1215**	**0.0772**	**0.0193**
	**FE**	**1.998**	**-0.0371**	**0.0674**	**0.0321**
**Outer race**	**BA**	**1.6077**	**-0.1731**	**0.0530**	**0.0012**
	**DE**	**1.1096**	**0.0433**	**0.1341**	**0.0367**
	**FE**	**1.5435**	**-0.0169**	**0.0968**	**0.0296**

## FLOST and IFLOST method

### ST and its inverse transform

S transform of a non-stationary signal can be defined as:

$$ST(\tau ,f)=\int_{-\infty }^{\infty }x(t)\frac{\left|f\right|}{\sqrt{2\pi }}e^{-\frac{{\left(\tau -t\right)}^{2}f^{2}}{2}}e^{-j2\pi ft}dt$$ (2)

Where *τ* and *t* are time variable, and *f* is frequency parameter. When S transform is integrated to time *τ*, then

$$\int_{-\infty }^{\infty }ST(\tau ,f)d\tau =\int_{-\infty }^{\infty }x(t)e^{-j2\pi ft}\left\{\int_{-\infty }^{\infty }h(\tau -t,f)d\tau \right\}dt$$ (3)

Where Gaussian window function $\int_{-\infty }^{\infty }h(\tau -t,f)d\tau =1$, and the Eq (3) is written as

$$\int_{-\infty }^{\infty }ST(\tau ,f)d\tau =\int_{-\infty }^{\infty }x(t)e^{-j2\pi ft}dt=X(f)$$ (4)

After Fourier inverse transform of *X*(*f*) is calculated, the original signal *x*(*t*) can be got.

$$x(t)=\int_{-\infty }^{\infty }X(f)e^{j2\pi ft}df=\int_{-\infty }^{\infty }\{\int_{-\infty }^{\infty }ST(\tau ,f)d\tau \}e^{j2\pi ft}df$$ (5)

### Fractional lower order S transform

The mechanical fault signal containing *α* stable distribution noise can be expressed as

y(t)=x(t)+v(t) (6)

Where *x*(*t*) is fault signal, *v*(*t*) is *SαS* distribution noise, *t* = 1,2,⋯,*N*. We apply fractional lower order moment to ST, and define fractional lower order S transform as

$$FLOST(\tau ,f)=\int_{-\infty }^{\infty }y^{<p>}(t)h(\tau -t,f)e^{-j2\pi ft}dt$$ (7A)

$$h(\tau -t,f)=\frac{\left|f\right|}{\sqrt{2\pi }}e^{-\frac{{\left(\tau -t\right)}^{2}f^{2}}{2}}$$ (7B)

Where <*p*> is *p* order moment operation, *p* is a real parameter(0 ≤ *p* < *α*/2)[26–28]. When *y*(*t*) is a real signal, *y*^<*p*>^(*t*) = |*y*(*t*)|^*p*−1^⋅*sign*[*y*(*t*)], $sign\left[y(t)\right]=\{\begin{array}{cc} 1 & y(t)>0\\ 0 & y(t)=0\\ -1 & \;y(t)<0 \end{array}$, and when *y*(*t*) is a complex signal, *y*^<*p*>^(*t*) = |*y*(*t*)|^*p*−1^⋅y^*^(*t*). *α* is index of *SαS* distribution, * demonstrate conjugate. *h*(*τ*−*t*,*f*) in Eq (7B) is a Gaussian window function related to the frequency, *f* is the frequency variable. *τ* and *t* are time variable, and *τ* is the center of the Gaussian window function.

The ST method is proposed based on short-time Fourier transform(STFT) and continuous wavelet transform (CWT). We assume that the Eq (7B) is only related to time parameter *t* and has nothing to do with *f*, then, the Eq (7A) will change as fractional lower order short time Fourier transform(FLOSTFT) [29], as shown in the Eq (8).

$$FLOSTFT(\tau ,f)=\int_{-\infty }^{\infty }y^{<p>}(t)h(\tau -t)e^{-j2\pi ft}dt$$ (8)

If we use mother wavelet function *w*(*t*,*f*) to replace *h*(*τ*−*t*,*f*)*e*^−*j*2*πft*^, then, the Eq (7A) becomes as fractional lower order continuous wavelet transform(FLOCWT)[30]

$$FLOCWT(\tau ,f)=\int_{-\infty }^{\infty }y^{<p>}(t)w(t,f)dt$$ (9)

Comparing the Eqs (7) and (9), then

$$FLOST(\tau ,f)=FLOCWT(\tau ,f)e^{-j2\pi ft}$$ (10)

### Inverse fractional lower order S transform

After FLOST in the Eq (7) is done integral of time *τ*, we can get

$$\begin{array}{l} \int_{-\infty }^{\infty }FLOST(\tau ,f)d\tau =\int_{-\infty }^{\infty }y^{<p>}(t)e^{-j2\pi ft}\left\{\int_{-\infty }^{\infty }h(\tau -t,f)d\tau \right\}dt\\ \;=\int_{-\infty }^{\infty }\bar{y}(t)e^{-j2\pi ft}\left\{\int_{-\infty }^{\infty }h(\tau -t,f)d\tau \right\}dt \end{array}$$ (11)

Where $\bar{y}(t)=y^{<p>}(t)$, the window function $\int_{-\infty }^{\infty }h(\tau -t,f)d\tau =1$, then

$$\int_{-\infty }^{\infty }FLOST(\tau ,f)d\tau =\int_{-\infty }^{\infty }\bar{y}(t)e^{-j2\pi ft}dt=\bar{Y}(f)$$ (12)

Where $\bar{Y}(f)$ is fractional lower order Fourier transform(FLOFT) of $\bar{y}(t)$. When the Eq (12) is done inverse operation, thus

$$\bar{y}(t)=\int_{-\infty }^{\infty }\bar{Y}(f)e^{j2\pi ft}df=\int_{-\infty }^{\infty }\{\int_{-\infty }^{\infty }FLOST(\tau ,f)d\tau \}e^{j2\pi ft}df$$ (13)

The Eq (13) shows that FLOFT of $\bar{y}(t)$ is the integral of FLOST time *τ*. Hence, we can get fractional lower order moment $\bar{y}(t)$ by calculating inverse Fourier transform of $\bar{Y}(f)$, then, we do inverse operation of $\bar{y}(t)$, and the original signal *y*(*t*) is got.

When *y*(*t*) is real signal, $\bar{y}(t)=y^{<p>}(t)={\left|y(t)\right|}^{p-1}\cdot sign\left[y(t)\right]$, according to the Eq (13), *y*(*t*) is written as

$$y(t)={\left[\bar{y}(t)\right]}^{\frac{1}{p-1}}sign[\bar{y}(t)],\;y(t)\;\mathrm{is}\;\mathrm{real}$$ (14)

Where $sign\left[\bar{y}(t)\right]=\{\begin{array}{cc} 1 & \bar{y}(t)>0\\ 0 & \bar{y}(t)=0\\ -1 & \;\bar{y}(t)<0 \end{array}$. When *y*(*t*) is complex signal, $\bar{y}(t)=y^{<p>}(t)={\left|y(t)\right|}^{p-1}\cdot y^{*}(t)$, letting $\bar{y}(t)=a_{t}+jb_{t}$, *t* = 1,2,⋯,*N*, then, *y*(*t*) can be got by solving equations $\bar{y}(t)=a_{t}+jb_{t}$, as shown in the Eq (15).

$$y(t)={\left|a_{t}\right|}^{\frac{1}{p}}{\left[1+{\left(b_{t}/a_{t}\right)}^{2}\right]}^{\frac{2}{p(p-1)}}sign(a_{t})-j{\left|b_{t}\right|}^{\frac{1}{p}}{\left[1+{\left(a_{t}/b_{t}\right)}^{2}\right]}^{\frac{2}{p(p-1)}}sign(b_{t}),\;y(t)\;\mathrm{is}\;\mathrm{complex}$$ (15)

## FLOST-TFF method

### ST-TFF method

Fourier transform of *x*(*t*) is the integral of ST time *τ*, which is expressed as:

$$X(f)=\int_{-\infty }^{\infty }x(t)e^{-j2\pi ft}dt=\int_{-\infty }^{\infty }S(\tau ,f)d\tau$$ (16)

To get or filter out the partial components in S time-frequency domain, filter strategy *F*(*τ*,*f*) can be employed as a data-adaptive time-frequency weight function, as shown in the Eq (17).

$$X^{\prime }(f)=\int_{-\infty }^{\infty }S(\tau ,f)F(\tau ,f)d\tau$$ (17)

Where *F*(*τ*,*f*) is real, *X*′(*f*) is the filtered Fourier transform of *x*(*t*), its inverse Fourier transform is written as

$$x^{\prime }(t)=\int_{-\infty }^{\infty }X^{\prime }(f)e^{j2\pi ft}df=\int_{-\infty }^{\infty }\int_{-\infty }^{\infty }S(\tau ,f)F(\tau ,f)e^{j2\pi ft}d\tau df$$ (18)

Where *x*′(*t*) is the filtered data.

### FLOST-TFF method

The traditional ST-TFR method fails under *α* stable distribution noise environment, and ST-TFF method based on S transform will degenerate. Hence, we use FLOST time-frequency method to replace ST method, and propose a fractional lower order S transform time-frequency filtering algorithm, the specific calculation process is as follows.

According to the Eq (12), we can get:

$$\bar{Y}(f)=\int_{-\infty }^{\infty }FLOST(\tau ,f)d\tau =\int_{-\infty }^{\infty }\bar{y}(t)e^{-j2\pi ft}dt$$ (19)

Each signal in FLOST time-frequency domain has a certain region, when we need to filter out noise or get some signals, we can select weight function *F*(*τ*,*f*) according to time and frequency characteristics of the signals, then

$${\bar{Y}}^{\prime }(f)=\int_{-\infty }^{\infty }FLOST(\tau ,f)F(\tau ,f)d\tau$$ (20)

**Fig 4:**
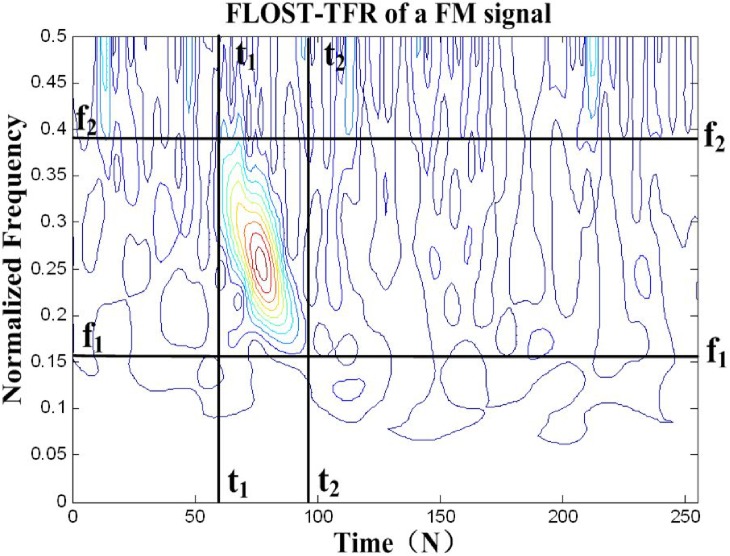
The passed domain of weight function *F*(*τ*,*f*).

Fig 4 is FLOST-TFR of a frequency modulation(FM) signal under *α* stable distribution noise environment. In order to filter out *α* stable distribution noise, we select the regions(*t*_1_ ≤ *τ* ≤ *t*_2_, *f*_1_ ≤ *f* ≤ *f*_2_) as time-frequency passed domain, and the other regions are regarded as noises.

After *F*(*τ*,*f*) is generated into the Eq (20), fractional lower order Fourier transform ${\bar{Y}}^{\prime }(f)$ is got. Then, we calculate inverse Fourier transform of ${\bar{Y}}^{\prime }(f)$, and fractional lower order moment ${\bar{y}}^{\prime }(t)$ is got, as shown in the Eq (21).

$${\bar{y}}^{\prime }(t)=\int_{-\infty }^{\infty }{\bar{Y}}^{\prime }(f)e^{j2\pi ft}df=\int_{-\infty }^{\infty }\{\int_{-\infty }^{\infty }FLOST(\tau ,f)F(\tau ,f)d\tau \}e^{j2\pi ft}df$$ (21)

Finally, let ${\bar{y}}^{\prime }(t)=a_{t}^{\prime }+jb_{t}^{\prime }$, *t* = 1,2,⋯,*N*, employing the Eqs 14 and 15, *y*′(*t*) is written as

$$y^{\prime }(t)=\{\begin{array}{l} {\left[{\bar{y}}^{\prime }(t)\right]}^{\frac{1}{p-1}}sign[{\bar{y}}^{\prime }(t)],\;y(t)\;is\;real\\ {\left|a_{t}^{\prime }\right|}^{\frac{1}{p}}{\left[1+{\left(b_{t}^{\prime }/a_{t}^{\prime }\right)}^{2}\right]}^{\frac{2}{p(p-1)}}sign(a_{t}^{\prime })-j{\left|b_{t}^{\prime }\right|}^{\frac{1}{p}}{\left[1+{\left(a_{t}^{\prime }/b_{t}^{\prime }\right)}^{2}\right]}^{\frac{2}{p(p-1)}}sign(b_{t}^{\prime }),\;y(t)\;is\;complex \end{array}$$ (22)

We can name the time-frequency filtering method in the Eqs (21 and 22) as fractional lower order S transform time-frequency filtering algorithm.

### Simplified FLOST-TFF method

We assume that a time-time function *y*_1_(*τ*,*t*) is expressed with p order moment of *y*(*t*) multiplying a Gaussian window function, as shown in the Eq (23).

$$y_{1}(\tau ,t)=y{\left(t\right)}^{<p>}\cdot e^{-\frac{{\left(\tau -t\right)}^{2}f^{2}}{2}}$$ (23)

Then, Fourier transform of *y*_1_(*τ*,*t*) is expressed as

$$Y_{1}(\tau ,f)=\int_{-\infty }^{\infty }y{\left(t\right)}^{<p>}\cdot e^{-\frac{{\left(\tau -t\right)}^{2}f^{2}}{2}}e^{-j\pi ft}dt$$ (24)

After multiplying $\frac{\left|f\right|}{\sqrt{2\pi }}$ on both sides of the Eq (24), then

$$\begin{array}{l} Y_{1}(\tau ,f)\cdot \frac{\left|f\right|}{\sqrt{2\pi }}=\int_{-\infty }^{\infty }y{\left(t\right)}^{<p>}\cdot e^{-\frac{{\left(\tau -t\right)}^{2}f^{2}}{2}}e^{-j\pi ft}dt\cdot \frac{\left|f\right|}{\sqrt{2\pi }}\\ \;=\int_{-\infty }^{\infty }y{\left(t\right)}^{<p>}\cdot \left\{\frac{\left|f\right|}{\sqrt{2\pi }}e^{-\frac{{\left(\tau -t\right)}^{2}f^{2}}{2}}\right\}e^{-j\pi ft}dt\\ \;=\int_{-\infty }^{\infty }y{\left(t\right)}^{<p>}\cdot h(\tau -t,f)e^{-j\pi ft}dt\\ \;=FLOST(\tau ,f) \end{array}$$ (25)

From the Eq (25), we know the relationship of *Y*_1_(*τ*,*t*) and *S*(*τ*,*t*) as

$$Y_{1}(\tau ,f)=\frac{\sqrt{2\pi }}{\left|f\right|}FLOST(\tau ,f)$$ (26)

We substitute the Eq (24) into the Eq (26), and compute Fourier inverse transform, then

$$y_{1}(\tau ,t)=\sqrt{2\pi }\int_{-\infty }^{\infty }\frac{FLOST(t,f)}{\left|f\right|}\cdot e^{j\pi ft}df$$ (27)

Assuming *τ* = *t*, then

$$\bar{y}(t)=y_{1}(t,t)=\sqrt{2\pi }\int_{-\infty }^{\infty }\frac{FLOST(t,f)}{\left|f\right|}\cdot e^{j\pi ft}df$$ (28)

According to the calculation process in section 4.2, we multiply weight function *F*(*t*,*f*) on both sides of the Eq (28), then, fractional lower order moment ${\bar{y}}^{\prime }(t)$ is got, as shown in the Eq (29).

$${\bar{y}}^{\prime }(t)=\sqrt{2\pi }\int_{-\infty }^{\infty }\frac{FLOST(t,f)F(t,f)}{\left|f\right|}\cdot e^{j\pi ft}df$$ (29)

When ${\bar{y}}^{\prime }(t)$ in the Eq (29) is substituted into the Eqs (21 and 22), the filtered *y*′(*t*) is got. Comparing with the method in section 4.2, the improved FLOST-TFF method in the Eq (29) has no time parameter *τ*, and no longer need to compute inverse Fourier transform. We can name the method in the Eq (29) as simplified fractional lower order S transform time-frequency filtering algorithm.

### Discrete calculation of the FLOST method

With *y*(*t*) sampling, the discrete *y*(*t*) can be written as *y*[*n*] = *y*(*nT*), *n* = 0,1,⋯,*N*−1, *T* is sampling period, and the corresponding sampling frequency *f*_*s*_ = 1/*T*, let frequency step as *f*_0_, *m* = −*M*/2,…,*M*/2−1 is discrete frequency range, *M* = *f*_*s*_/*f*_0_. Then, the discrete FLOST form in the Eq (7) can be written as

$$FLOST[l,m]=\sum_{n=0}^{N-1}\bar{y}[n]\frac{\left|m\right|}{\left|M\right|\sqrt{2\pi }}e^{-\frac{m^{2}{\left(l-n\right)}^{2}}{2M^{2}}}e^{-\frac{j2\pi mn}{M}}$$ (30)

Where $\bar{y}[n]=y^{<p>}[n]$ is the discrete fractional p order moment of *y*[*n*]. FLOST in the Eq (30) is defined based on fractional p order moment of the signal in time-domain, according to the definition of frequency ST based on Fourier transform in [12], then, ST employing fractional lower order Fourier transform is defined as

$$FLOST(\tau ,f)=\int_{-\infty }^{\infty }\bar{Y}(u+f)e^{-\frac{2\pi^{2}u^{2}}{f^{2}}}e^{j2\pi u\tau }du$$ (31)

Where $\bar{Y}(u+f)$ is Fourier transform of *y*^<*p*>^(*u* + *f*). The Eq (31) change as

$$FLOST[l,m]=\sum_{q=0}^{M-1}\bar{Y}[\frac{m+q}{M}]e^{-\frac{4\pi^{2}q^{2}}{m^{2}}}e^{-\frac{j2\pi ql}{M}}$$ (32)

According to fractional lower order Fourier transform in the Eq (19), we can get the discrete calculation formula, as shown in (33), and its discrete inverse fractional lower order Fourier transform is shown in (34).

$$\bar{Y}[m]=\sum_{l=0}^{N-1}FLOST[l,m]$$ (33)

$$\bar{y}[n]=\frac{1}{M}\sum_{m=-M/2}^{M/2-1}\bar{Y}[m]e^{\frac{j2\pi mn}{M}}$$ (34)

Substituting the Eq (33) into the Eq (34), then

$$\bar{y}[n]=\frac{1}{M}\sum_{m=-M/2}^{M/2-1}\sum_{l=0}^{N-1}FLOST[l,m]e^{\frac{j2\pi mn}{M}}$$ (35)

Assuming discrete adaptive time-frequency weight function as *F*[*l*,*m*], then, the discrete filter strategy with *F*[*l*,*m*] is expressed as

$${\bar{y}}^{\prime }[n]=\frac{1}{M}\sum_{m=-M/2}^{M/2-1}\sum_{l=0}^{N-1}FLOST[l,m]F[l,m]e^{\frac{j2\pi mn}{M}}$$ (36)

Similarly, we can get the discrete equation of the simplified FLOST-TFF method in section 4.3, as shown in (37).

$${\bar{y}}^{\prime }[n]=\sum_{m=-M/2}^{M/2-1}\frac{FLOST[l,m]F[l,m]}{\left|m\right|}e^{\frac{j2\pi mn}{M}}$$ (37)

When ${\bar{y}}^{\prime }[n]$ in (37) is substituted into the Eqs (21 and 22), the filtered original signal is got.

### The steps of FLOST-TFF method

Step 1:Computing *FLOST*[*l*,*m*] employing the Eq (30) or the Eq (32).
Step 2: Selecting appropriate weight function *F*[*l*,*m*] according to FLOST spectrum of the signals.
Step 3: Computing IFLOST with substituting *FLOST*[*l*,*m*] and *F*[*l*,*m*] into the Eq (36) or (37), and getting fractional p order moment ${\bar{y}}^{\prime }[n]$.
Step 4: Performing inverse operation of ${\bar{y}}^{\prime }[n]$ employing the Eqs (21 and 22), and getting the restored original signal *y*′[*n*].

## Simulations

We design the following experiments to test the proposed FLOST method, FLOST-TFF algorithm, the existing ST method and ST-TFF algorithm. The test signals *y*_1_(*n*) and *y*_2_(*n*) are selected as

$$y_{1}(n)=x_{1}[1:128]+x_{2}[129:256]+x_{3}[160:165]+x_{4}[180:185]+v(n)=x(n)+v(n)$$ (38)

$$y_{2}(n)=x_{5}(n)+x_{6}(n)+v(n)$$ (39)

Where *x*_1_(*n*) = cos(2πn*36/256), *x*_2_(*n*) = cos(2πn*10/256), *x*_3_(*n*) = cos(2πn*80/256), *x*_4_(*n*) = cos(2πn*80/256), $x_{5}(n)=e^{-a{\left(n-N_{1}\right)}^{2}+jc{\left(n-N_{1}\right)}^{2}+j\omega (n-N_{1})}$, $x_{6}(n)=e^{-a{\left(n-N_{2}\right)}^{2}+jc{\left(n-N_{2}\right)}^{2}+j\omega (n-N_{2})}$, *a* = 0.004, *c* = −0.025, *ω* = 1.72, *N*_1_ = 80, *N*_2_ = 180, *n* = 0,1,⋯,256, *v*(*n*) is Gaussian noise or *SαS* distribution noise. *SNR* can be used if *v*(*n*) is Gaussian noise, but when *v*(*n*) is *SαS* distribution noise, *SNR* is no more applicable. Hence, generalized signal noise ratio(*GSNR*) is given, which is expressed as *GSNR* = 10 log_10_ {*E*[|*x*(*n*)|^2^]/*γ*^*α*^}.

### Simulation comparisons of the ST-TFR and FLOST-TFR method

**Fig 5:**
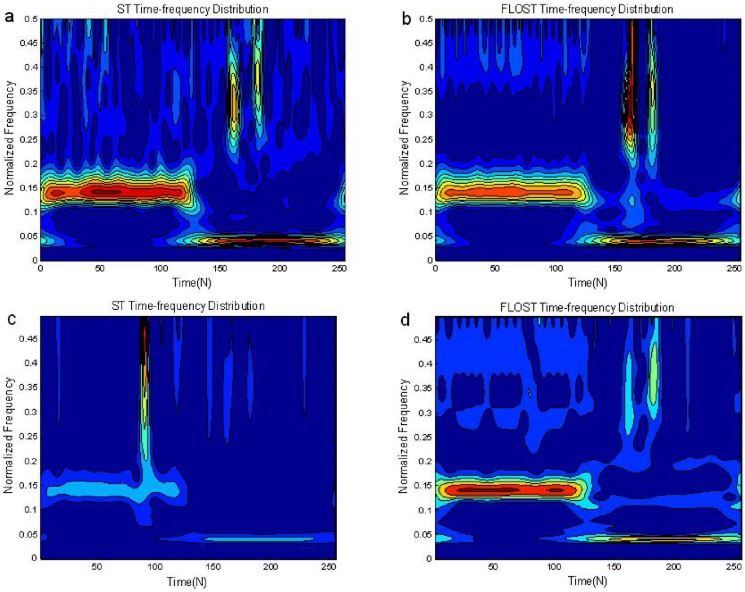
Time-frequency representations of *y*_1_(*n*) employing the ST and FLOST methods (a) ST Time-frequency representation under Gaussian noise environment (b) FLOST Time-frequency representation under Gaussian noise environment (c) ST Time-frequency representation under *SαS* distribution noise environment (d) FLOST Time-frequency representation under *SαS* distribution noise environment.

In this simulation, we select *y*_1_(*n*) as the test signal. When *v*(*n*) is Gaussian noise, let *SNR* = 10*dB* and *p* = 0.8, and when *v*(*n*) is *SαS* distribution noise, let *α* = 1.1, *p* = 0.1, *GSNR* = 18*dB*. We compare the proposed FLOST time-frequency algorithm and the existing ST time-frequency algorithm under Gaussian noise and *SαS* stable distribution noise environment, respectively. The experimental results are shown in Fig 5A–5D. Fig 5(A) and Fig 5(B) are the time-frequency representations of ST and FLOST algorithms under Gaussian noise environment, the figures show that both methods can better demonstrate time-frequency distribution of the signal *y*_1_(*n*).

The time-frequency representation of *y*_1_(*n*) employing ST method is shown in Fig 5(C) under *SαS* stable distribution noise environment, which shows that ST time-frequency method fail, and its time-frequency representation is incorrect. The improved FLOST time-frequency method can better reveal the time-frequency distribution of *y*_1_(*n*), as shown in Fig 5(D). Therefore, the FLOST time-frequency method has better performance than ST time-frequency method, and is robust.

### Simulation comparisons of ST-TFF and FLOST-TFF algorithm

**Fig 6:**
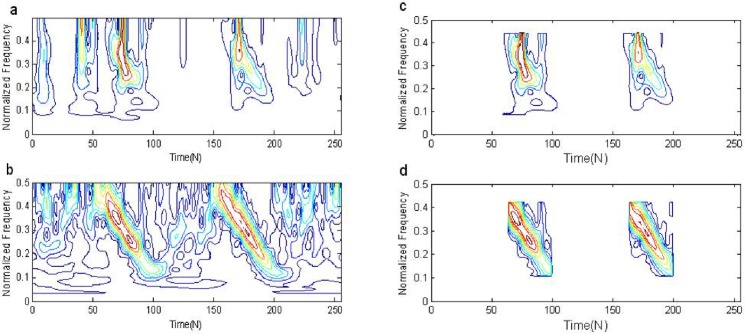
Time-frequency filtering employing ST and FLOST under *SαS* distribution noise environment (a) Time-frequency representation of *y*_2_(*n*) employing ST method (b) Time-frequency representation of *y*_2_(*n*) employing FLOST method (c) The filtered time-frequency representation employing the ST-TFF method (d) The filtered time-frequency representation employing the Eq (37).

In this simulation, *y*_2_(*n*) is selected as the test signal, *v*(*n*) is *SαS* distribution noise, *α* = 1.1, *p* = 0.1, and *GSNR* = 18*dB*. We apply ST-TFF and FLOST-TFF methods to filter out *SαS* distribution noise, the simulations are shown in Fig 6A–6D. Fig 6(A) is ST time frequency representation of *y*_2_(*n*), and Fig 6(B) is time frequency representation of *y*_2_(*n*) employing FLOST-TFR algorithm. Time frequency distribution of *y*_2_(*n*) which is filtered by ST-TFF method is shown in Fig 6(C), and the filtered time frequency representation of *y*_2_(*n*) employing the FLOST-TFF method is shown in Fig 6(D). The experimental results show that ST-TFR and ST-TFF methods based on ST degrade under *SαS* distribution noise environment, but the FLOST-TFR method can effectively suppress *SαS* distribution noise and clearly demonstrate time frequency distribution of *y*_2_(*n*), and FLOST-TFF algorithm has good performance to filter out the noise. Hence, the FLOST-TFF algorithm is better than the ST-TFF method.

**Fig 7:**
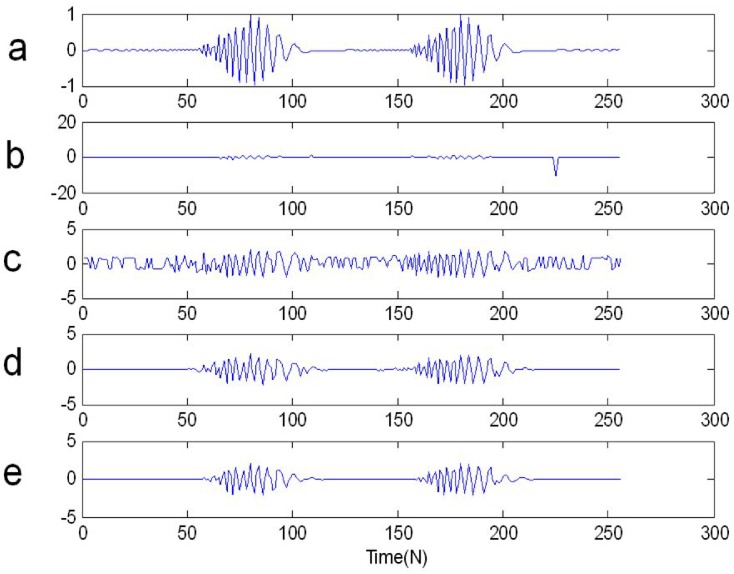
The real waveforms in time domain (a) Two FM signals (b) Two FM signals + *SαS* distribution noise (c) *y*_2_(*n*) IST to time domain after ST time frequency filtering (d-e) *y*_2_(*n*) IFLOST to time domain after time frequency filtering with the Eqs (36) and (37), respectively.

In Fig 7 we show from top to bottom: the real waveform of two FM signals in time domain, two FM signals contaminated by *SαS* distribution noise, *y*_2_(*n*) IST to time domain from time frequency domain after ST time frequency filtering, *y*_2_(*n*) IFLOST to time domain after time frequency filtering employing the Eqs (36) and (37), respectively. The experimental results show that the estimated *y*_2_(*n*) employing ST-TFF method in Fig 7(C) has larger deviation, but *y*_2_(*n*) estimated by FLOST-TFF method in Fig 7D and 7E is alike with the original signal in Fig 7(A), which reveal the preponderance of the improved FLOST-TFF algorithm.

### MSE comparisons of ST-TFF and FLOST-TFF method under different *α* and *GSNR*

In this simulation, we select *y*_2_(*n*) as the test signal, and define mixed mean square error (*MSE*) as

$$MSE=\frac{1}{2K}\sum_{k=1}^{K}{\left({\hat{x}}_{5}-x_{5}\right)}^{2}+\frac{1}{2K}\sum_{k=1}^{K}{\left({\hat{x}}_{6}-x_{6}\right)}^{2}$$ (40)

*K* is the number of Monte-Carlo experiment, *x*_5_ and *x*_6_ are two original LFM signals, ${\hat{x}}_{5}$ and ${\hat{x}}_{6}$ are their estimations employing ST-TFF or FLOST-TFF.

**Fig 8:**
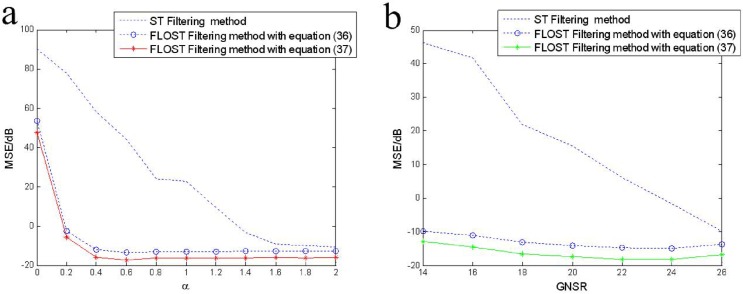
The mixed MSE comparisons of the ST-TFF method and FLOST-TFF algorithm employing the Eqs (36) and (37) (a) *GSNR* = 18*dB*, *α* changes from 0 to 2 (b) *α* = 1.2, *GSNR* changes from 14dB to 26dB.

Let *GSNR* = 18*dB*, *p* = 0.1, *K* = 20. When *α* changes from 0 to 2, we apply ST-TFF and FLOST-TFF methods to restore *x*_5_ and *x*_6_ in different *α*, the mixed *MSEs* are given in Fig 8(A). The result shows that the mixed *MSEs* of FLOST-TFF method employing (36) are stable in −15*dB* when *α* changes from 0.4 to 2, and the mixed *MSEs* employing (37) are about −17*dB*, however, the mixed *MSEs* employing ST-TFF method change from 60*dB* to −14*dB*. Hence, the estimation error of the proposed FLOST-TFF algorithm is lower than the ST filtering algorithm under *α* stable distribution noise.

Let *α* = 1.2, *p* = 0.1, *K* = 20. When *GSNR* changes from 14*dB* to 26*dB*, the mixed *MSEs* of ST and FLOST methods are compared under *α* stable distribution noise environment. The experimental simulations are given in Fig 8(B), the result shows that *MSEs* of the improved FLOST-TFF methods employing the Eqs (36) and (37) are lower than that of the ST-TFF method, and which are stable in −10*dB* to −18*dB*. Especially, when *GSNR* < 18*dB*, the advantage of FLOST-TFF methods is more obvious.

### Applications of FLOST time-frequency filtering to machine fault diagnosis

**Fig 9:**
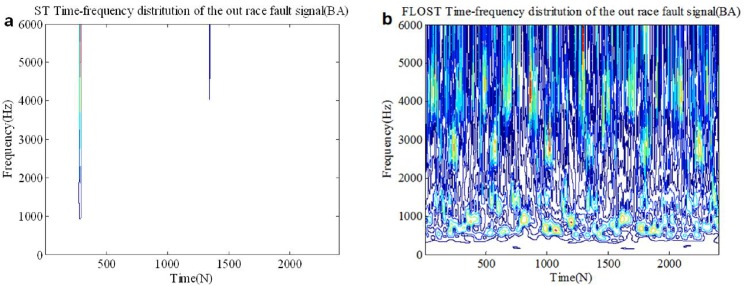
Time-frequency representation of the out race fault signal in BA under *SαS* distribution noise environment (a)Time-frequency representation of the out race fault signal in BA employing ST-TFR method (b) Time-frequency representation of the out race fault signal in BA employing FLOST-TFR method.

**Fig 10:**
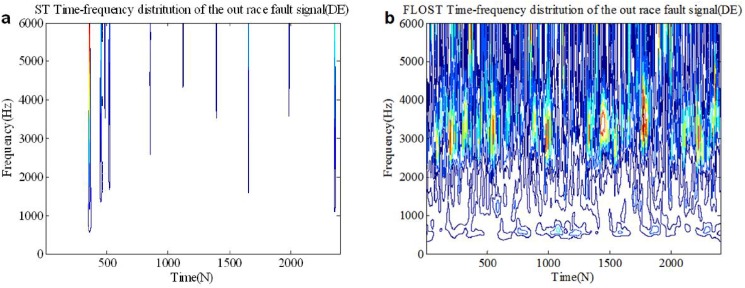
Time-frequency representation of the out race fault signal in DE under *SαS* distribution noise environment (a)Time-frequency representation of the out race fault signal in DE employing ST-TFR method (b) Time-frequency representation of the out race fault signal in DE employing FLOST-TFR method.

**Fig 11:**
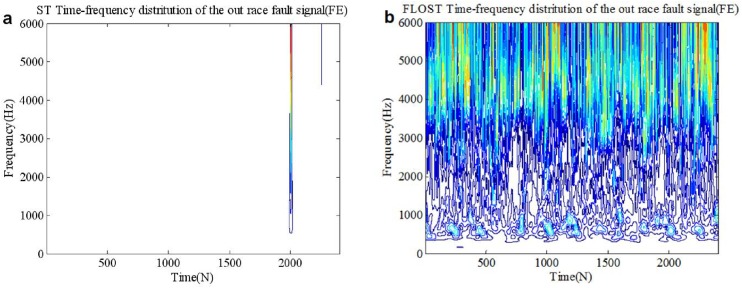
Time-frequency representation of the out race fault signal in FE under *SαS* distribution noise environment (a)Time-frequency representation of the out race fault signal in FE employing ST-TFR method (b) Time-frequency representation of the out race fault signal in FE employing FLOST-TFR method.

In this simulation, the experimental signals adopt the out race bearing fault in BA, DE and FE from S3 Mat in section 2, we select 0.2 seconds data as test signal, then *N* = 2400. *SαS* distribution noise (*α* = 1.1, *GSNR* = 15*dB*) is added as the actual working environment background noise. The ST-TFR and FLOST-TFR methods are applied to extract fault feature of the out race fault signals, the simulation results are shown in Fig 9, Fig 10 and Fig 11. Fig 9(A) and Fig 9(B) are time frequency representations of the out race fault signal in BA employing the ST-TFR and FLOST-TFR methods, respectively. The time frequency representations of the out race fault signal in DE and FE are shown in Fig 10 and Fig 11, respectively. The results show that the time frequency distributions employing the ST-TFR method in Fig 9(A), Fig 10(A) and Fig 11(A) are incorrect, but the FLOST-TFR method has good performance, as shown in Fig 9(B), Fig 10(B) and Fig 11(B), and we can know that the fault vibration interval is about 30 ms, the fault characteristic frequency is about 33.333 Hz, transient harmonic vibration component is about 3300 Hz.

**Fig 12:**
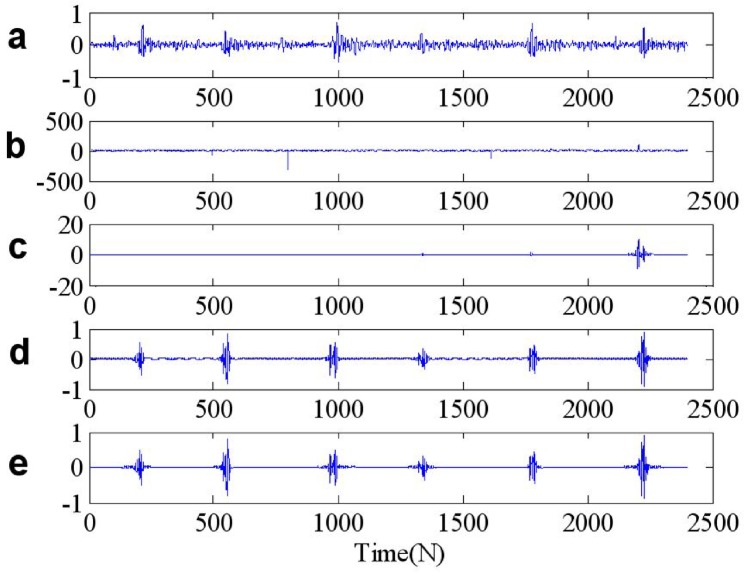
Time waveforms of two chirp signals (a) The out race fault signal in BA (b) The out race fault signal in BA + *SαS* distribution noise (c) The filtered out race fault signal in BA employing ST-TFF method (d-e) The filtered out race fault signal in BA employing the Eqs (39) and (40), respectively.

**Fig 13:**
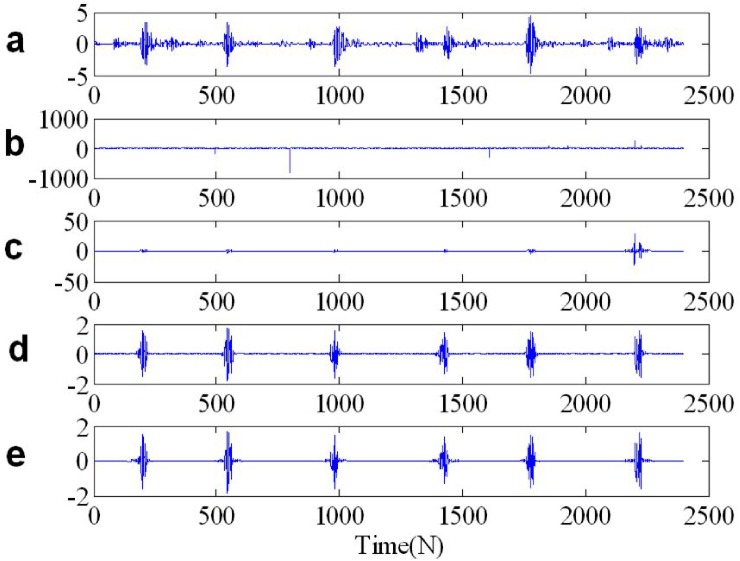
Time waveforms of two chirp signals (a) The out race fault signal in DE (b) The out race fault signal in DE + *SαS* distribution noise (c) The filtered out race fault signal in DE employing ST-TFF method (d-e) The filtered out race fault signal in DE employing the Eqs (39) and (40), respectively.

**Fig 14:**
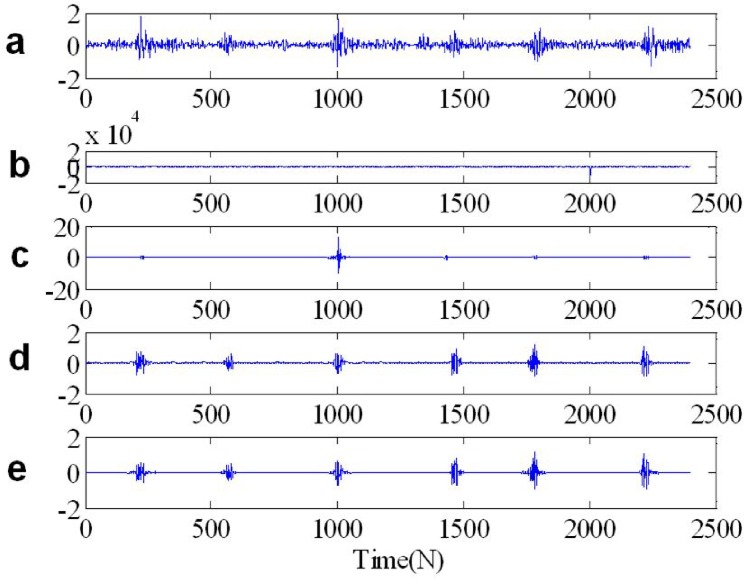
Time waveforms of two chirp signals (a) The out race fault signal in FE (b) The out race fault signal in FE + *SαS* distribution noise (c) The filtered out race fault signal in FE employing ST-TFF method (d-e) The filtered out race fault signal in FE employing the Eqs (39) and (40), respectively.

We apply the ST-TFF and FLOST-TFF methods to restore the original signal from *SαS* distribution noise, the simulation results are shown in Fig 12, Fig 13 and Fig 14. In Fig 12, Fig 13 and Fig 14 we show from top to bottom: the out race fault signal in BA, DE and FE, respectively, the out race fault signal in BA, DE and FE contaminated *SαS* distribution noise, respectively, the filtered out race fault signal IST to time domain from time frequency domain, the out race fault signal inverse IFLOST to time domain after time frequency filtering with the Eqs (36) and (37), respectively. The Fig 12 results show that the estimated out race BA fault signal employing ST-TFF method has larger deviation in Fig 12(C), but the estimated out race BA fault signal in Fig 12D and 12E is similar to the original signal in Fig 12(A) because of employing FLOST-TFF method. Hence, the existing ST-TFF method is invalid, but the proposed FLOST-TFF method can effectively suppress *SαS* distribution noise and restore the original signal from time-frequency domain, which has a certain toughness. Similarly, Fig 13 and Fig 14 reveal the same conclusion. Hence, the FLOST-TFF method has better performance to recovering the original signal from *α* stable distribution noise.

The time frequency filtering technology which takes advantage of time-frequency localized spectra of the data provides an adaptive-filtering method for the non-stationary signals. The time frequency filtering method applies an adaptive weighting function to separate out the signals from the noise. The higher weighting parts localize the regions which are expected to be the signal components, and the lower weighting parts attenuate the noise in the time-frequency domain. The inverse transform of time frequency representation is used to reconstruct the original signals.We can set the parameter *p* according to characteristic index of *α* stable distribution noise, smaller *α* is, the smaller *p*. When *p* = 2, the FLOST-TFR method degenerates into the ST-TFR method, and IFLOST change into IST method, then, the corresponding FLOST-TFF method degenerates into ST-TFF method. Hence, the improved FLOST-TFR and FLOST-TFR methods are generalized algorithm.

## Conclusions

This paper proves that bearing fault signals belong to *α* stable distribution with the range of *α* from 1 to 2. The time-frequency analysis method is a key tool for machinery fault diagnosis, which can be used to identify the constituent components and time variation of the fault signals. We have presented a fractional lower order S transform time frequency distribution algorithm applied for *SαS* distribution noise environment, which can effectively suppress *SαS* distribution noise, and work under low *GSNR*. We apply the FLOST time frequency method to analyze the test signal in Gaussian noise environment and *SαS* stable distribution noise. It is proved that the FLOST-TFR method has better performance and toughness than the existing ST-TFR method. The FLOST-TFR method has no cross interference comparing with the secondary FLO-PWVD time-frequency distribution method, which remedies lack of phase information comparing with the fractional lower order continuous wavelet transform method. FLOST time frequency filtering method is proposed to separate the bearing out race fault signals from *α* stable distribution noise in this paper. We also apply the FLOST-TFR method to analyze time frequency distribution of the fault signal and extract its fault feature. The FLOST-TFF method is employed to filter *SαS* distribution noise in time frequency domain, and inverse FLOST is used to restore the original signal. It is verified that the mixed *MSE* of the FLOST-TFF method is smaller than that of the existing ST-TFF method, also its performance is better than ST-TFF method. In some practical applications, when the mechanical bearing fault signals or noises belong to *α* stable distribution process(1 < *α* < 2), we can extract the fault features from time frequency representation of the mechanical fault signals employing FLOST-TFR method, filter out *α* stable distribution noise employing FLOST-TFF method, and restore the original fault signal with inverse FLOST. Furthermore, we can also use the FLOST-TFR and FLOST-TFF methods to analyze the signals even when the mechanical bearing fault signals or the noises belong to Gaussian distribution process(*α* = 2), just setting up reasonable parameter *p* according to characteristic index of *α* stable distribution noise is needed.

## Supporting information

S1 Mat The data is the bearing normal signal from the case western reserve university data center [25], which is used as the experimental signals in “Bearing fault signals” section. (MAT)

S2 Mat The data is the bearing ball fault signal from the case western reserve university data center [25], which is used as the experimental signals in “Bearing fault signals” section. (MAT)

S3 Mat The data is the bearing out race fault signal from the case western reserve university data center [25], which is used as the experimental signals in “Bearing fault signals” and “Applications of FLOST time-frequency filtering to Machine Fault Diagnosis” section. (MAT)

S4 Mat The data is the bearing inner race fault signal from the case western reserve university data center [25], which is used as the experimental signals in “Bearing fault signals” section. (MAT)
